# Border crossings and connections

**DOI:** 10.1186/s40694-024-00182-5

**Published:** 2024-08-22

**Authors:** Martin Weinhold

**Affiliations:** Berlin, Germany

**Keywords:** Fungi, Art, Science, Communication, Collaboration, Biotechnology, Biomaterials

## Abstract

From 30 September 2023 to 7 January 2024, the Nobel Prize Museum in Stockholm presented the show *Fungi—In Art and Science.* For the exhibition, an alliance of scientists, artists, and designers was brought together that overcame all the alleged borders between the disciplines, between the scientific and the creative world. This special exhibition is the starting point to take on a tour where it is about crossing borders and growing connections when working with fungi. My interview partners represent perfectly the different angles from which you can take a look onto the kingdom of fungi. There is the person without previous knowledge but with a profound artistic understanding who got mesmerized by the subject-matter, which he didn’t realize it existed before—Karl-Johan Cottman. There is the scientist, being knee-deep in fungi matter who discovered the arts for an extension of her scientific understanding—Vera Meyer. And last but not least there is the person living passionately for the arts who found fungi mesmerizing for both art creation and progressive/sustainable production—Phil Ross. So, there are three threads weaving one fungal fabric. Have fun reading it!

## The exhibition in Stockholm

**Fig. 1:**
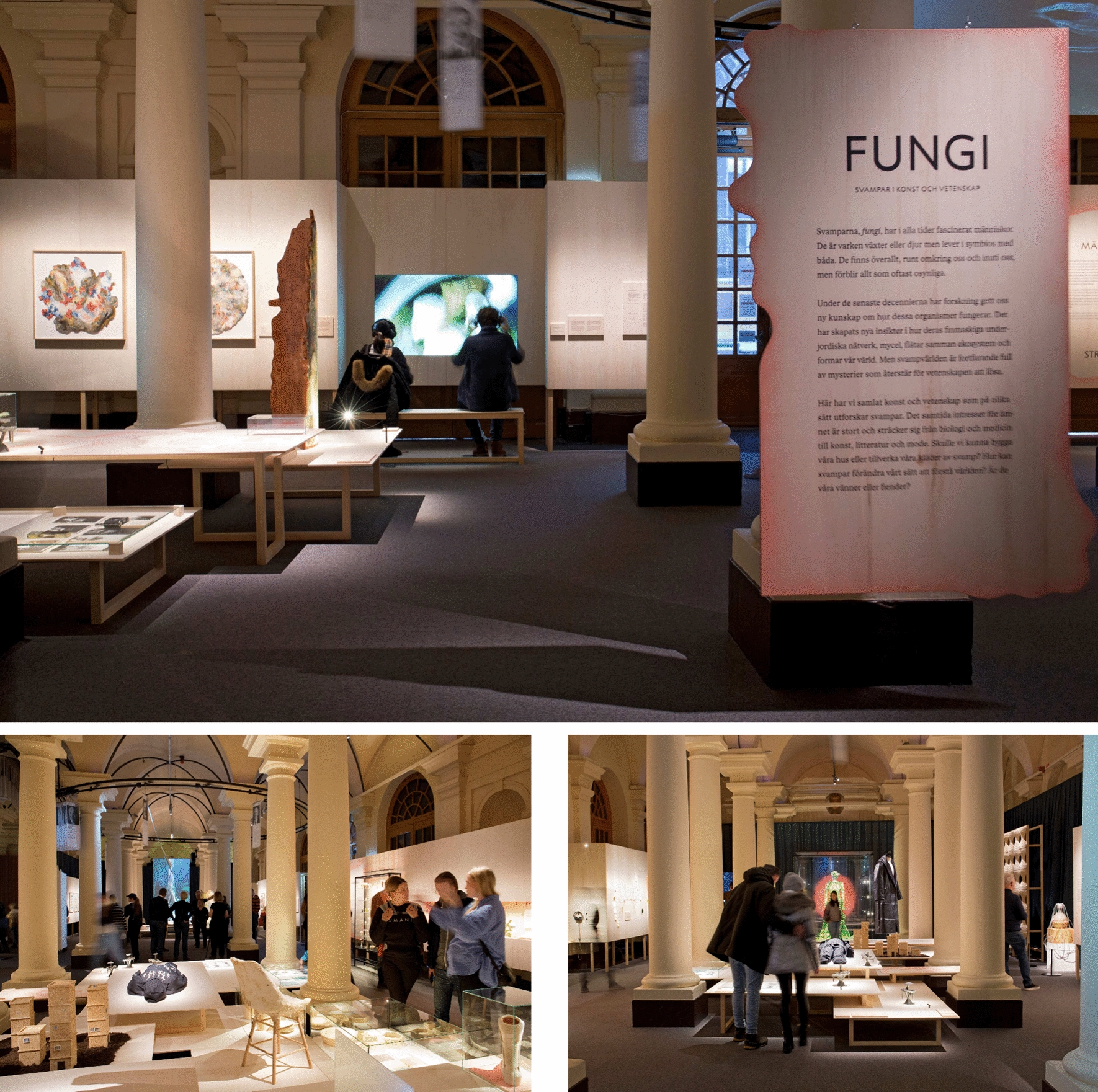
The exhibition *Fungi—In Art and Science* at the Nobel Prize Museum in Stockholm

An image gallery of Nobel laureates is guiding the visitor from the museum’s entrance towards the building’s interior. The portraits of honoured men and women move slowly and by jerks along a track system on the ceiling, move towards the rear room and turn there in an infinite loop. At their turning point, a presentation board welcomes the visitor to the exhibition *Fungi—In Art and Science*. From there the show expands symmetrically into a space between old columns. The face sides catch the visitor’s eye quickly with a stage costume once worn by pop artist Björk glowing in shiny green on one side and a tall video installation with a rotating tree trunk on the other (Fig. 1).

The Nobel Prize Museum created a highly topical mosaic of fungi references placed in a dignified historical building. The manifold structured exhibition ground is made of bright wood. With its various levels and surfaces, it gives the room an organic rhythm. Exhibits from the realms of art, science, and design change constantly, convey knowledge about the species fungi, which is neither plant nor animal. Material samples are on display, screens show the growth of fungi or present short videos in which scientists explain basic concepts of fungal research (Fig. 1). Benches are placed in front of the monitors; many visitors remain there sitting for a long time, listening mesmerized. The exhibition feels like a hike, taking you from subject to subject, guiding you through a forest of discoveries. Through playful transitions the show easily connects exhibits from different fields thus helping us to understand that we are looking at an interconnected whole.

## The curator intrigued by fungi

**Fig. 2:**
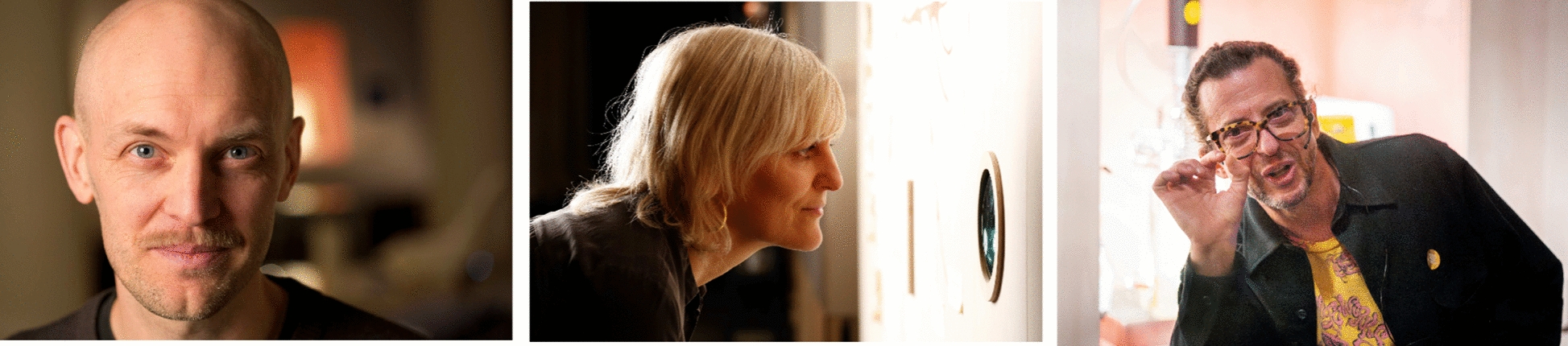
Karl-Johan Cottman (left) and Phil Ross (right) at the opening evening in September 2023 ( © Nobel Prize Museum). Vera Meyer (middle) visiting the show in November 2023

Karl-Johan Cottman (Fig. 2) works as a curator at the Nobel Prize Museum. For the interview we meet in the museum’s beautiful building in the old town of Stockholm. Light snow is falling, the Christmas market attracts crowds of tourists in front of the main entrance. It’s Monday, the museum is closed, but it’s busy inside nonetheless. School classes are led through the exhibition, maintenance people take care of minor repairs or replace blown light bulbs. During the weekend the temporary show *Fungi—In Art and Science* was packed with people from morning until closing hour.

In November 2022 Karl-Johan Cottman had joined the Nobel Prize Museum’s team. It was in his very first year that he got involved in curating a show about the world of fungi together with his colleague Magnus af Petersens. Why did they choose to exhibit fungi? Karl-Johan Cottman explains that the decision was made intuitively, based on a shared gut feeling. Everyone in the team had the impression that fungi attract more and more attention and therefore deserved to be in the spotlight. “We saw examples in a lot of different fields, like art, design, fashion, medicine, biotechnology, and so on, where a lot of people are now working with fungi. Obviously, there was something going, what with everyone being interested in the same subject. This is what we got intrigued by. Fungi also seemed to be a good match for a cross-disciplinary approach, which is at the core of the Nobel Prize Museum with its special mix ranking from chemistry to literature. And we were then translating this first amazement into an exhibition.”

At first the concept was designed for a rather small and focused show. Karl-Johan Cottman remembers how in the very beginning he thought “There can’t be that much about fungi.” Smilingly he adds: “Now, in hindsight, I think it should have been clear that it is such a complex subject-matter. If you were asked to make an exhibition about animals—everybody would know this is huge. But for the fungal kingdom it is something you still have to realise.”

The cross-disciplinary idea for the show was there early on. The team’s actual starting point for exhibits was the *Fermenting Futures Project*, a series of art pieces done by Anna Dumitriu and Alex May, using the CRSIPR/Cas9 technique for modifying yeasts, which are unicellular fungi. Their work had an excellent link to the museum, since in 2020 the Nobel Prize in Chemistry was awarded to Emmanuelle Charpentier and Jennifer Doudna for discovering the CRISPR/Cas9 genetic scissors. In February 2023 the network of contacts for the exhibition had grown quickly. Karl-Johan Cottman called it a „growing mycelium of fungi-involved people.” This is also when it became clear that they could no longer think in terms of a small exhibition. The spectrum was already much larger than they had expected.

Phil Ross came into play in the spring of 2023 and envisioned a more experimental take on his presentation as the museum’s team originally had in mind. He also suggested to bring in Vera Meyer right away. When the two of them brought up the idea to show a working bioreactor, Karl-Johan Cottmann had to admit that he had no clue what that might be. In June 2023 they all met in Berlin and visited Vera Meyer's lab, the Department of Applied and Molecular Microbiology. There Karl-Johan Cottman saw a bioreactor for the first time and was thrilled. Phil Ross and Vera Meyer suggested to reveal more of the actual process, an idea which the curators welcomed: “Magnus and I liked the idea to focus on the biotechnology itself and not just on its outcome. For us it was great to show the process and production which usually is hidden behind lab doors. We wanted to depict the complexity and the scientific background, not just display the finished object like in a design show.”

Having limited space in a hall lined with pillars was a challenge for the show’s design, a challenge brilliantly mastered by the curating team and their exhibition designer Birger Lipinski. The display tables and boards are made out of light wood, wood being an obvious choice for the subject of fungi, as Karl-Johan Cottman points out. The varying levels of display tables filling the room were conceived by the designer to resemble the undulating bottom of the forest. Then, by putting objects on these different levels, they tried to use the height according to the respective exhibit’s size, in order to make them equal to the viewer’s eye. What they wanted to stress with their presentation was that everything is connected.

Karl-Johan Cottman mentions the rhizome as another metaphor in relation to the design of the exhibition levels: “Rhizome is a term in biology but also has been used in philosophy. What it describes is a horizontal structure where everything is connected to everything. And it is also underneath, so that things pop up from the rhizome. But they are not individual entities, they are all part of the whole. Birger used that kind of work because it is related to the philosophy of a holistic view. He wanted to create a design that suggests by its irregular shape: hierarchies are not that apparent. Different platforms pop up in different spaces and they have an equal value, no matter where they show up.”

With the show running now for two months, it is also a good moment for a first interim balance. According to Karl-Johan Cottman, one of the museum’s main objectives with this kind of temporary theme exhibition is to increase the number of local visitors. In this respect alone, the exhibition is already a great success: “Before we had a ratio of 90 percent tourists and only about 10 percent people from Stockholm. Now with this show we came close to 50:50 on weekend days.”

When asked about how people respond to the fungi exhibition, Karl-Johan Cottman takes a moment to think. Then he replies: “One of my observations when I am in the exhibit space is that people spend a lot of time there. They really sit down and watch and listen. People seem to find something they like. They find details they remember. Even the most basic facts about fungi are completely new to people and in turn create curiosity when they read or hear about it. So, what happened to them is what happened to us—it was a revelation to learn about fungi.” His personal perspective has changed as well, after being immersed in the subject for an intense period of time: “When I go out walking in the woods now, I have a different view I sense more of nature’s interconnectedness. Also, once you’ve learned about fungi, you never again think a handful of soil is empty.”

## The scientist exploring the arts

There is the scientist Vera Meyer (Fig. 2). Immersed in fungal research for more than 25 years, internationally connected, invited to speak on conferences from California to Shanghai. The list of her publications is a long one. And there is Vera Meyer’s relation to the arts. It is an unusual liaison in which passion meets factual conviction. Passion when it comes to creating own artworks and a factual conviction that art can achieve so much to convey scientific ideas. Within the last 300 years, natural sciences and the arts and humanities were separated artificially, that’s what Vera Meyer is saying repeatedly. It is on us to bring them back together—this is an important part of her vision. Both the sciences and the arts are creative processes in order to “understand the world, depict the world, change the world.” She is certain we will need cross-disciplinary approaches if we want to find solutions for the burning issues of our time. Which is why, in 2018, she opened up her Department at Technische Universität Berlin for collaborations with artists. And has never again closed the door on them.

For the interview we sit in a small restaurant in Stockholm after having spent the whole day in the exhibition *Fungi—In Art and Science*. The show has left a strong impression on Vera Meyer and still reverberates within.

How could she not have been thrilled when Phil Ross called her in June 2023 and told her about the plans for the exhibition? Because she, too, wants to make science tangible! Vera Meyer speaks about her early experience with press and media: “Whenever I gave interviews in the first years of my research in Berlin, I was trying to make things understandable through data and facts. I explained everything within the scientific context. Only to realise in the end that it remained way too abstract for most people. By using the arts as a medium and a bridge, you can reach people differently. Make people see, smell, touch! Make knowledge perceptible and the senses will open a portal into the sciences. It made me so happy when I learned that the curators in Stockholm had called the exhibition *Fungi—In Art and Science* and therefore did not limit the show on fungi in science.”

Artistic creation and manual work were helpful for Vera Meyer’s own research work. When she began sculpting with fungal material, she noticed the remarkable stability of the tinder fungus which led her to important conclusions: “I can stand on it and it doesn’t break, I can screw something into it and it remains, I can even put a nail into it. The fruiting body is shock-resistant, lightweight, and even water-repellent. To deal with this material artistically helped me to understand it scientifically. It also had a tangible effect on our research. A couple of years ago we decided in our Department to collect all different kinds of mushrooms in the Berlin-Brandenburg region. The goal was to analyse which of these fungi could be cultivated in the laboratory on residual waste streams from agriculture and forestry. We collected about 70 to 80 species, among them the tinder fungus. When we later applied for a grant on the subject of building with fungi, I said: ‘Let’s take the tinder fungus as the central topic of our experiment because this species has such outstanding material properties.’ I wouldn't have made this decision if I hadn't worked with the fungus in the studio beforehand.”

**Fig. 3:**
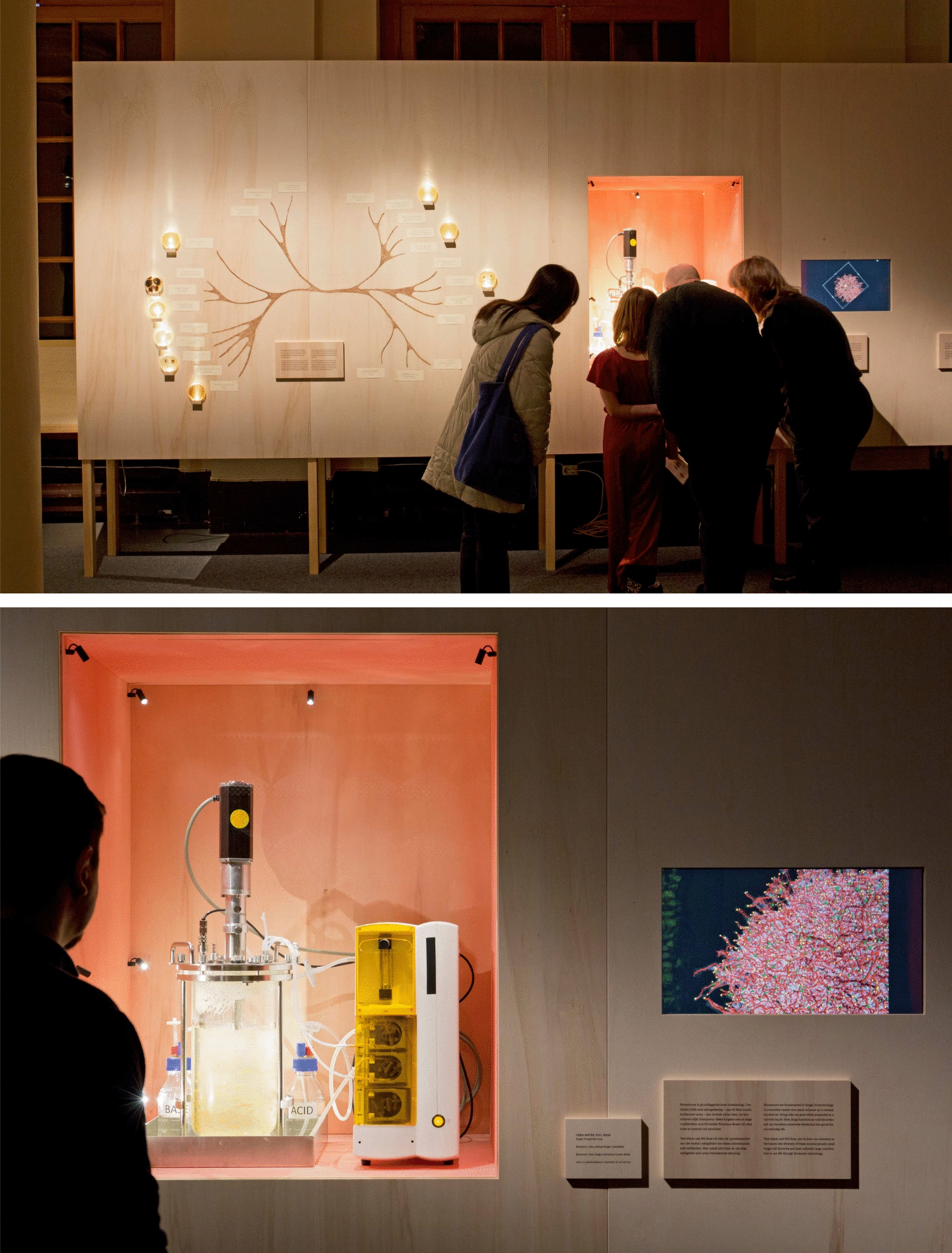
*Fungal Transformer* by Vera Meyer and Phil Ross (2023). Through a phylogenetic tree, Vera Meyer and Phil Ross have visualized the evolutionary relationships among some industrial fungi and products they already produce for us. Bioreactors are fundamental to fungal biotechnology. In controlled environmental vessels that reach volumes up to several hundred m3, living cells can grow while suspended in a nutritive liquid. Here, fungi functions as “cell factories” and can transform renewable feedstocks into goods for our everyday life. Vera Meyer and Phil Ross aim to draw our attention to the beauty and diversity of these microscopically small fungal cell factories and their infinitely large contribution to our life through bioreactor technology

In Vera Meyer’s view it will be of crucial importance to use fungi in bioreactors on an industrial scale if we want to succeed with the transformation of our petroleum-based economy into a sustainable one. To illustrate this aspect, she and Phil Ross developed the installation *Fungal Transformer* for the exhibition. In their arrangement four different views complement each other: we see the fungus’ nutrient solution in an operating bioreactor, we see the structure of *Aspergillus niger* in an animated X-ray tomography, find its classification within a phylogenetic tree and finally see a tiny freeze-dried fungal pellet being isolated from the bioreactor (Fig. 3).

Vera Meyer stresses the importance of naming the 24 industrially useable fungi in the phylogenetic tree with their precise species titles. “They deserve having their names mentioned!” Sweeping generalisations are still too common, she says, shaking her head, and continues: “Nobody would call an exhibition just ‘Animals’. Just as there are millions of animal species, there are also millions of fungal species—and of course, they are neither all good nor bad. Some fungi can produce useful and important products for our daily life, others make people sick or threaten to destroy entire harvests. But when it comes to sustainable solutions for the future, there’s a lot we can learn from fungi. For instance, how we as humans can accept the planetary limits. Fungi are doing this very successfully since millions of years by collaborating across species’ boundaries.”

Collaboration is also a central topic for Vera Meyer. Given the overwhelming amount of knowledge in our present time, she thinks the era of universal scholars like Alexander von Humboldt is over. In collectives and teams, however, the creative potentials could be united and unleashed. Which is what she practices in her Department successfully. Vera Meyer is rather wondering why it hasn’t long since found its way into other areas of science. Isn’t it obvious that different perspectives on the same phenomenon lead to different understandings and therefore different approaches to solutions? For Vera Meyer, this is a form of biodiversity: “When you bring together people from various backgrounds in work teams, when you respect, use and integrate their differing views so that the solutions found are ultimately sustainable. Cross-disciplinary collaborations are not just an opportunity but a necessity if we want to quickly solve the pressing problems of our time.”

For the Biotech community working with fungi, she is optimistic. There she can see a growing number of alliances where architects, artists, designers, and biotechnologists collaborate closely. But it would be as important to disseminate the knowledge into society. In an ideal world, Vera Meyer would like to enable each and everybody to work with fungal technology. She already detects signs for a trend into that direction: more people are baking their own bread, micro-breweries are springing up, kombucha is being made at home. Why shouldn’t people be able to grow their own furniture in ten years’ time with the help of locally available fungi? Curiosity and the courage to experiment together—that’s Vera Meyer’s wish for the future. For her that’s what the exhibition in Stockholm motivates us to do: Operate with fungi!

## The artist in science and business

Artist, citizen scientist, entrepreneur—where do you place Phil Ross exactly? He laughs when I ask the question and responds that he has given up placing himself. Phil Ross (Fig. 2) is a wanderer between worlds by conviction. We conduct the interview by phone in January 2024. Phil Ross is sitting in the Santa Cruz mountains, I’m in Berlin. There is static noise and crackling, sometimes the line breaks off; it hisses like in the days of the first Atlantic cable.

Phil Ross’ relationship with mushroom-forming fungi began in the kitchen, as an extension of cooking. He was employed in a restaurant located in the wooded valley of the Hudson River, New York State. Mushrooms practically grew on his doorstep. Their different flavours mesmerised him, and they lent so much sophistication to his vegetarian dishes! So he started growing mushrooms in order to supply his kitchen. Next, fungi entered his artwork. In 1997 he inoculated a huge sculpture, a replica Boeing 747 built from garbage, with oyster mushroom spawn. During the 5-week exhibition at Gallery 16 in San Francisco, the fruiting bodies of the tremendously multiplying fungus ate the sculpture. Now what? Was he a cook, a mushroom grower, an artist? It was all connected, intertwined and affecting one another.

Phil Ross was already well established in the bio-art scene when he started experimenting with fungal mycelium. He was keen on developing completely new materials. The astonishing results quickly aroused the interest of multinational companies, so much so that it led him to set up the company MycoWorks. The leather alternative, which MycoWorks has been producing on an industrial scale since 2023, also attracted the attention of curator Karl-Johan Cottman, who invited Phil Ross to participate in the exhibition *Fungi—In Art and Science* at the Nobel Prize Museum. He convinced the curator’s team in Stockholm to go beyond the mere display of Fine Mycelium samples but to instead emphasise the process of developing mycelium-derived materials. Asking Vera Meyer from the TU Berlin to join the exhibition project was the other suggestion he made to Karl-Johan Cottman. He describes how his and Vera Meyer’s joint contribution to the Nobel Prize Museum’s show came into being: “It’s been a fantasy of mine for almost twenty years to do exhibits in science museums, particularly in the biology section. One example of what I found very interesting and what only few people know: Almost all of our vitamin C is grown in bioreactors. It doesn’t come from citrus fruit. Similarly, many of our cancer medicines are made with bioreactors. So many elements of our world are grown inside these reactors and I always wanted to make that visible in an aesthetic way that is interesting to the audience.“

For the exhibition Vera Meyer and Phil Ross talked a lot about the history of bioreactors, the history of industrial fungi. They were wondering how to make this instrument and this process apparent. Phil Ross explains their train of thought: “How do we visualise this very standard method, this synthetic space humans created for fungi and other microorganisms? A space from which we derive such phenomenal elements of the natural world that we are able to isolate and expand, to grow in volume. You can think of this access to the dimension of fungi as a portal. Once we created the bioreactor, we created a way to access the elements of fungal space and micro space. It has just begun to open and the things that are already spilling out are like bounty, manna and jewels. That’s why Vera and I wanted to describe how the fungal world opens up when you use this instrument, and we came up with the idea to showcase an operating bioreactor as the central element.”

Because Phil Ross came into the fungal science world as an artist, he could be more daring in this field. As he points out: “I don’t have a job to lose, I don’t have all the responsibility of somebody working in a scientific institution. I have the freedom of the arts.” In the course of founding MycoWorks he met with mycologists and geneticists. Phil Ross encountered them as an entrepreneur when his company was figuring out the industrial production of fungal materials. Very soon he noticed that their perspective on fungi was entirely different from his. Their focus in almost all cases was to kill fungal microorganisms or to prevent them from growing—getting rid of mould on the wall or avoiding crop losses in forestry or agriculture caused by fungal infestation. But there was surprisingly little expertise about fungal genomes with respect to what great substances they can produce. For Phil Ross, however, this is where today’s “Arctic territories” lie: a promising *terra incognita* where discoverers will surely be rewarded. He calls it “an entirely new dimension of possibilities” where the great deeds of modernity can be accomplished. Phil Ross can easily envision mycelium grown spaceships or mobile phones. It may take another decade or two, he concedes. The microorganisms need to be developed, as well as the structures for this new fungal economy—institutions, funding, and thousands of young people ready to launch into fungal-related research. If that happens, almost anything would be possible.

## Epilogue

“Today I am actually even more mesmerized by fungi compared to the very beginning of my scientific career.” Vera Meyer says. “In those days I still was focussing very much on one specific species, having one research aspect in mind. Now, through the arts, this spectrum has broadened immensely. I have learned through fungi way more than I ever would have thought or expected. Very importantly, I also realized that in nature positive interaction predominates. Collaboration and cohesion is what made nature so strong. Organisms survive because they collaborate. A beautiful example out of the exhibition is the video installation *Breathing with the Forest* created by the London-based experiential art collective *Marshmellow Laser Feast*. It gives only one pivotal information: you see the ecosystem surrounding a tree in the Colombian Amazon. It takes only this single sentence, everything else is being clarified through the artistic rendering: the symbiosis of the tree with fungi and other domains of life and how they exchange nutrients. This piece of work is breathtakingly beautiful. The viewer learns: this is one organic being. For me the installation also indicates that we urgently need to reconsider the concept of individuality. Yes, there is this one tree and several fungal species. But as a matter of fact, they survive because they cooperate. We as humans are colonized by billions of microbial cells from bacteria and fungi and they all live in symbiosis with us. We thus exist as metaorganisms, we are not individuals but live and survive as communities.”

## Data Availability

No datasets were generated or analysed during the current study.

